# Rolling Bearing Fault Diagnosis Method Based on SWT and Improved Vision Transformer

**DOI:** 10.3390/s25072090

**Published:** 2025-03-27

**Authors:** Saihao Ren, Xiaoping Lou

**Affiliations:** Key Laboratory of the Ministry of Education for Optoelectronic Measurement Technology and Instrument, Beijing Information Science and Technology University, Beijing 100192, China; saihaoren@163.com

**Keywords:** rolling bearings, fault diagnosis, variable operating conditions, synchronized wavelet transform (SWT), CAA attention mechanism, Vision Transformer

## Abstract

To address the challenge of low diagnostic accuracy in rolling bearing fault diagnosis under varying operating conditions, this paper proposes a novel method integrating the synchronized wavelet transform (SWT) with an enhanced Vision Transformer architecture, referred to as ResCAA-ViT. The SWT is first applied to process raw vibration signals, generating high-resolution time–frequency maps as input for the network model. By compressing and reordering wavelet transform coefficients in the frequency domain, the SWT enhances time–frequency resolution, enabling the clear capture of instantaneous changes and local features in the signals. Transfer learning further leverages pre-trained ResNet50 parameters to initialize the convolutional and residual layers of the ResCAA-ViT model, facilitating efficient feature extraction. The extracted features are processed by a dual-branch architecture: the left branch employs a residual network module with a CAA attention mechanism, improving sensitivity to critical fault characteristics through strip convolution and adaptive channel weighting. The right branch utilizes a Vision Transformer to capture global features via the self-attention mechanism. The outputs of both branches are fused through addition, and the diagnostic results are obtained using a Softmax classifier. This hybrid architecture combines the strengths of convolutional neural networks and Transformers while leveraging the CAA attention mechanism to enhance feature representation, resulting in robust fault diagnosis. To further enhance generalization, the model combines cross-entropy and mean squared error loss functions. The experimental results show that the proposed method achieves average accuracy rates of 99.96% and 96.51% under constant and varying load conditions, respectively, on the Case Western Reserve University bearing fault dataset, outperforming other methods. Additionally, it achieves an average diagnostic accuracy of 99.25% on a real-world dataset of generator non-drive end bearings in wind turbines, surpassing competing approaches. These findings highlight the effectiveness of the SWT and ResCAA-ViT-based approach in addressing complex variations in operating conditions, demonstrating its significant practical applicability.

## 1. Introduction

Rolling bearings play a crucial role in rotating machinery, with their condition having a direct influence on the performance of the entire system [1,2]. Statistics show that approximately one-third of failures in electromechanical drive systems and motor systems are attributed to rolling bearing faults. Therefore, timely fault diagnosis of rolling bearings is crucial for maintaining the performance and reliability of rotating machinery [3].

Intelligent fault diagnosis algorithms generally comprise sensor signal acquisition, feature extraction, and fault classification techniques [4]. In signal acquisition, accelerometers are typically employed to capture bearing vibration signals. Time–frequency analysis is commonly utilized for feature extraction, as it analyzes signals in both the time and frequency domains, providing a clear representation of the relationship between frequency and time for non-stationary signals [5]. This approach transforms vibration signals into the time–frequency domain, extracts fault features, and then feeds them into fault classification algorithms. Prominent fault classification techniques currently rely on machine learning and deep learning methods [6].

Various machine learning methods, including artificial neural networks, decision trees, and support vector machines, have been utilized for fault diagnosis [7]. However, these methods heavily depend on expert knowledge for feature extraction and selection, have limited feature representation capabilities, and lack generalization ability, and their shallow structures hinder the capture of deep features and complex relationships in the data [8].

In contrast, deep learning methods offer superior feature extraction and processing capabilities, as well as improved generalization and model transferability, leading to their widespread adoption in fault diagnosis applications [9].

He et al. [10] introduced a residual network that addresses the issue of gradient vanishing in backpropagation by incorporating skip connections, thus enhancing the model’s ability to extract deep features. Zhang et al. [11] tackled the issue of inadequate feature extraction in deep learning models by utilizing the Short-Time Fourier Transform (STFT) to transform raw signals into a two-dimensional time–frequency representation, which is then input into an improved convolutional neural network (CNN) for efficient classification. Tao et al. [12] introduced an unsupervised fault diagnosis approach for rolling bearings. This method combines time–frequency information fusion via wavelet packet decomposition with an enhanced maximum mean discrepancy algorithm, leading to enhanced accuracy and robustness. Guo [13] proposed a fault diagnosis approach for complex scenarios, using variational mode decomposition, sensitive component analysis, and time–frequency feature extraction, along with a hybrid deep learning model, demonstrating high diagnostic accuracy and robustness. Peng et al. [14] employed data fusion techniques and residual neural networks for efficient rolling bearing fault diagnosis. Despite the excellent performance in feature extraction and classification accuracy, these methods still exhibit limitations in fault diagnosis performance under complex multi-condition scenarios. Variations in operating conditions significantly affect bearing vibration signals, and traditional methods often struggle to handle these variations, leading to decreased classification accuracy [15].

To improve fault classification under complex conditions, Wen et al. [16] proposed a TCNN (ResNet-50) model that leverages transfer learning. By transforming raw signals into RGB images and utilizing ResNet-50 as a feature extractor, this model achieved an accuracy of up to 99.99%, outperforming both traditional and deep learning methods. However, its performance is highly dependent on the pre-trained model and may struggle with significant distribution shifts under variable operating conditions. To address the challenge of label imbalance in rolling bearing fault diagnosis, Que et al. [17] introduced an inter-class feature transfer mechanism. This approach mitigates the impact of outliers and enhances classification accuracy. Nevertheless, its performance may be constrained when handling highly non-stationary signals. Lei [18] proposed an unsupervised graph transfer network that integrates multi-scale and multi-structure information for fault diagnosis across varying conditions. By combining node feature extraction, multi-scale convolutional layers, attention mechanisms, and Graph Neural Networks (GNNs), this method improves adaptability to complex scenarios. However, it does not fully exploit fine-grained time–frequency features, which are crucial for early fault detection.

While these methods have advanced fault diagnosis performance, they still face key limitations. One major challenge is their limited ability to capture high-resolution time–frequency features, which are crucial for early fault detection. Additionally, they often lack an effective mechanism to integrate both local and global fault representations, restricting their adaptability to varying operating conditions.

Motivated by these challenges, this paper proposes a fault diagnosis method for rolling bearings based on the synchronized wavelet transform (SWT) and ResCAA-ViT, aiming to improve fault classification under varying operating conditions. Unlike conventional methods that either rely solely on time–frequency analysis or employ standalone neural networks, our approach uniquely integrates SWT’s high-resolution time–frequency features with the ResCAA-ViT hybrid architecture. The SWT offers superior energy concentration and time–frequency resolution, enabling more precise fault detection, particularly under non-stationary conditions. The ResCAA-ViT model further enhances this by integrating local feature extraction with the CAA attention mechanism and global feature capture via a Vision Transformer, providing a more informative and comprehensive fault representation. Unlike ResNet-based models, which heavily depend on pre-trained CNN architectures, our approach improves adaptability to varying operating conditions. Compared to feature transfer and GNN-based methods, our dual-branch model not only enhances robustness but also explicitly captures both fine-grained local features and global fault structures, resulting in more precise and interpretable diagnostics.

The innovations and contributions of this study are as follows:

(1) This study innovatively applies the Synchrosqueezed Wavelet Transform (SWT) to process raw vibration signals, generating high-resolution time–frequency representations. By compressing and reordering wavelet coefficients in the frequency domain, the SWT enhances energy concentration, resulting in clearer and more focused time–frequency maps. This approach enables the effective capture of instantaneous changes and localized features of the signal, which are often difficult to detect using conventional techniques such as the Continuous Wavelet Transform (CWT) or Short-Time Fourier Transform (STFT). As a result, the SWT significantly improves the fault diagnosis capabilities of the model, offering superior resolution and sensitivity in detecting faults, particularly under complex and non-stationary conditions.

(2) A novel ResCAA-ViT hybrid architecture is proposed, combining the strengths of CNNs and Vision Transformers (ViTs) for enhanced fault diagnosis. The left branch integrates a residual network with the CAA (Channel Attention Aggregation) attention mechanism, which combines local feature extraction, strip convolution, and adaptive channel weighting to improve feature representation. The right branch utilizes a Vision Transformer to capture global dependencies through its self-attention mechanism. Transfer learning accelerates model training in small-sample scenarios, while multi-loss constraints ensure effective network convergence. The proposed model demonstrates superior classification accuracy and generalization ability, outperforming traditional methods in complex fault detection tasks.

(3) In the context of rolling bearing operating under variable load and noisy conditions, experimental validation was conducted using the CWRU dataset. The results show that the proposed method surpasses the alternative approaches in both fault diagnosis accuracy and generalization ability. Furthermore, validation using a real-world dataset from the non-drive end bearing of wind turbines further substantiates the method’s potential for practical industrial applications.

## 2. Related Theories and Methods

### 2.1. Synchrosqueezed Wavelet Transform

The Synchrosqueezed Wavelet Transform (SWT) is a time–frequency analysis method that offers high resolution in both time and frequency. This is accomplished by compressing and rearranging the wavelet transform coefficients along the frequency dimension [19]. The application of the SWT to the time–frequency analysis of vibration signals typically involves three main steps, as outlined below:

Step 1: Continuous Wavelet Transform (CWT).

The signal s(t) is first transformed using the CWT, which produces wavelet coefficients $W_{s}\left(a,b\right)$. These coefficients represent the signal in both time and frequency domains.

Step 2: Instantaneous Frequency Calculation.

The instantaneous frequency $\omega_{s}\left(a,b\right)$ is calculated by analyzing the phase of the wavelet coefficients. This step extracts the frequency information associated with the signal’s time-varying characteristics.

Step 3: Synchrosqueezing Transformation.

The synchrosqueezing step refines the time–frequency representation by compressing wavelet coefficients according to their instantaneous frequencies. This results in a more accurate and concentrated time–frequency map.

The synchrosqueezed wavelet transform is defined by the following formula:

(1) $$T_{s}\left(\omega_{\ell },b\right)=\Delta \omega^{-1}\sum_{a_{k}:\left|\omega \left(a_{k},b\right)-\omega_{\ell }\right|}W_{s}\left(a_{k},b\right)a^{-\frac{3}{2}}{\left(\Delta a\right)}_{k}$$

### 2.2. Loss Function

Commonly used loss functions include cross-entropy loss and mean squared error (MSE) loss. Cross-entropy loss quantifies the difference between predicted values and true labels, while MSE loss measures the discrepancy between the outputs of two branches.

The overall loss function used in this study is as follows:

(2) $$L_{total}=L_{\mathrm{Cross-entropy}1}+L_{\mathrm{Cross-entropy}2}+\lambda L_{\mathrm{MSE}}$$

(3) $$L_{\mathrm{MSE}}=\frac{1}{2}\left({\left(L_{\mathrm{Cross-entropy}1}-L_{\mathrm{Cross-entropy}2}\right)}^{2}\right)$$

In the equation, $L_{total}$ denotes the total loss, $L_{\mathrm{Cross-entropy}}$ represents the cross-entropy losses for the two branches, $L_{\mathrm{MSE}}$ represents the mean squared error (MSE) loss between the two cross-entropy losses, and λ denotes the weight parameter. This multi-loss constraint combines the cross-entropy losses from the two branches with their MSE loss, thereby enhancing the model’s consistency and collaborative learning capabilities.

### 2.3. CAA Module

The Context Anchor Attention (CAA) module, discussed in this study, is derived from the Poly Kernel Inception Network (PKINet) proposed by Cai et al. As a crucial component of PKINet, the CAA module is intended to capture long-range contextual dependencies and enhance the representation of crucial features [20].

**Figure 1:**
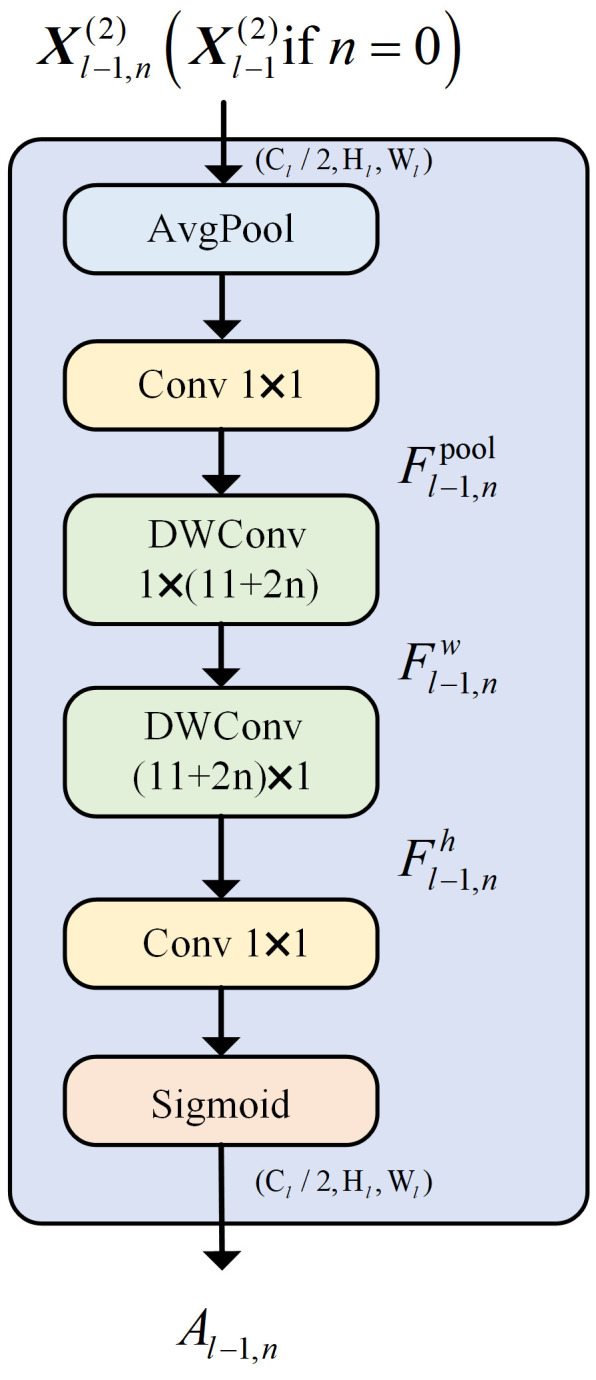
CAA module.

The CAA module consists of key stages: local feature extraction, strip convolution, attention weight generation, and feature enhancement, as shown in Figure 1. Initially, average pooling is applied to the input feature map, followed by a 1 × 1 convolution to produce local region features. The module then captures long-range contextual information using strip convolutions. A vertical convolution ($1\times k_{b}$) extracts vertical features, and a horizontal convolution ($k_{b}\times 1$) captures horizontal features. Strip convolutions reduce the number of parameters compared to standard $k_{b}\times k_{b}$ convolutions while maintaining performance. The kernel size $k_{b}$ is dynamically adjusted based on the network depth, allowing the receptive field to grow progressively. The CAA module generates attention weights using a 1 × 1 convolution followed by a Sigmoid activation. These attention weights are multiplied element-wise with the input features, and residual connections are used to preserve the original features. This process enhances the feature representation, as important regions are emphasized. Finally, a 1 × 1 convolution is applied to reduce the channel dimensions, producing the final output.

By integrating local feature extraction with global contextual modeling, the CAA module enhances feature representation while maintaining a low computational cost. Specifically, the CAA module improves feature representation in two key aspects: first, contextual enhancement, where the module extracts local features through average pooling followed by 1 × 1 convolutions and then uses lightweight strip depth-wise convolutions to capture both vertical and horizontal contextual information. This approach enables the model to focus on important features at multiple scales, improving its robustness in handling complex tasks. Second, the attention mechanism, where the generated attention weights, obtained through a Sigmoid activation applied to the convolution results, highlights key regions of the feature map. This mechanism effectively guides the model to prioritize the most relevant areas of the input, allowing it to disregard irrelevant information and perform better in complex scenarios. Moreover, the CAA module’s strip convolutions offer a significant computational advantage over conventional 2D convolutions by reducing the number of parameters while maintaining the model’s ability to capture long-range dependencies. The dynamic adjustment of the kernel size based on network depth further expands the receptive field, thereby enhancing the quality of contextual modeling.

The Context Anchor Attention (CAA) module outperforms standard attention mechanisms like Squeeze-and-Excitation (SE) and self-attention by efficiently capturing long-range dependencies with strip convolutions. Unlike SE, which uses global average pooling and 1 × 1 convolutions, CAA first extracts local features and then applies strip convolutions to capture both vertical and horizontal contextual information. This approach enhances feature representation at multiple scales, improving robustness in complex tasks. Additionally, strip convolutions reduce computational complexity compared to self-attention mechanisms, which require pairwise attention between all features, making CAA both more effective and computationally efficient.

In the proposed model architecture, the CAA attention module is placed both before and after Layer 4 of ResNet50. This dual placement enhances feature extraction at different stages of the network. The CAA module before Layer 4 improves local feature extraction and contextual enhancement, strengthening the representation of low-level features. Placing it after Layer 4 refines these features, enabling the model to capture more complex, high-level dependencies. This design ensures the effective integration of both local and global contextual information, facilitating multi-scale feature fusion. Consequently, the model captures intricate patterns and improves robustness across varying conditions.

### 2.4. Vision Transformer Model

**Figure 2:**
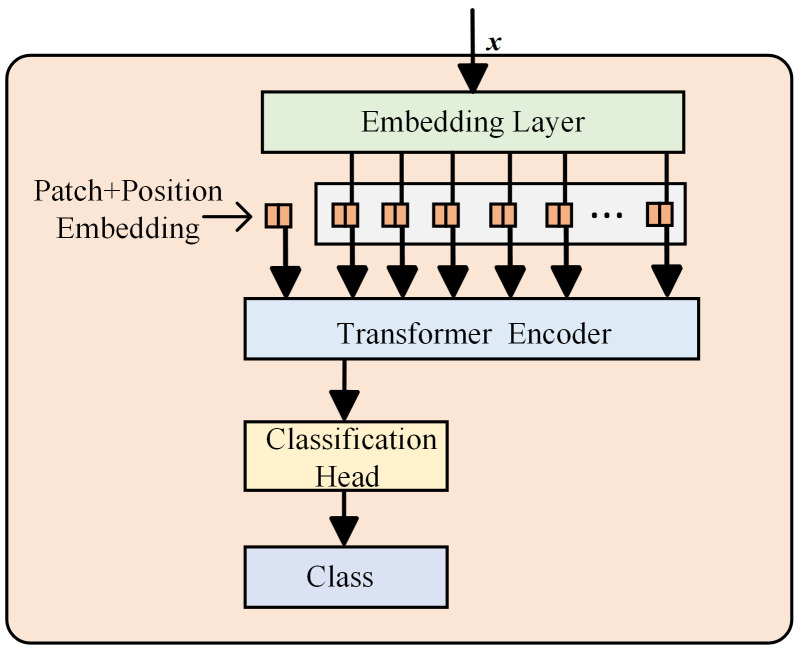
ViT model architecture.

Dosovitskiy et al. introduced the Vision Transformer (ViT) model, which partitions an image into sequences of patches and embeds them into a Transformer for image classification and feature extraction [21]. The ViT model consists of an embedding layer where the image is divided into patches, which are flattened and projected into a higher-dimensional space; patch and position embeddings that add spatial information to preserve relationships; Transformer encoder layers that process patch embeddings and capture global dependencies; a classification head typically based on the Class Token; and a final class module that maps the output to class labels for classification. The architecture of the ViT model is depicted in Figure 2.

The proposed ResCAA-ViT’s right branch incorporates the Vision Transformer (ViT) model, which leverages its self-attention mechanism and global receptive field to capture complex, long-range dependencies in the time–frequency spectrogram. The integration of ResNet and ViT in this branch represents a novel combination of convolutional and transformer-based architectures, enabling the model to benefit from the unique strengths of both. ResNet, with its residual connections and ability to learn hierarchical feature representations, efficiently captures local, low-level features in the initial layers. In contrast, ViT excels at modeling global contextual information and high-level features. By combining these two architectures, the proposed model effectively captures multi-scale features, enhancing its robustness and performance across a wide range of tasks and complex multi-dimensional data, such as time–frequency spectrograms.

### 2.5. Pretraining and Transfer Learning

Pre-trained networks are deep learning models with parameters learned from extensive training on large-scale datasets, resulting in optimal configurations of model parameters and hyperparameters. Training large-scale neural networks typically demands extensive labeled data and significant computational resources. Transfer learning mitigates this challenge by leveraging parameters or learned representations from pre-trained models, significantly improving model performance, particularly in data-scarce scenarios.

In rolling bearing fault diagnosis, where labeled data are often limited, transfer learning provides an effective solution to enhance model accuracy. By utilizing parameters from models pre-trained on large-scale datasets, transfer learning effectively alleviates overfitting caused by limited samples while expediting model convergence and optimization. Pei et al. [22] proposed a rolling bearing fault diagnosis method based on transfer learning, employing ResNet-50 as the backbone network. They integrated a Wasserstein Generative Adversarial Network with Gradient Penalty (WGAN-GP) to generate synthetic samples and utilized Multi-Kernel Maximum Mean Discrepancy (MK-MMD) to minimize the distribution discrepancy between the source and target domains. The experimental results showed that this method attained a fault diagnosis accuracy of 96.58% on the CWRU dataset. Similarly, Chen et al. [23] froze the parameters of the Conv1 layer and the first three residual layers in ResNet-50, transferring them to a Deep Transfer Convolutional Neural Network (DTCNN) for fault classification. Their experimental results indicated that freezing these layers significantly enhanced training efficiency, mitigated overfitting on small datasets, and improved model classification accuracy. These findings suggest that selectively freezing pre-trained layers can effectively boost model generalization and classification performance in rolling bearing fault diagnosis. ResNet-50, pre-trained on the ImageNet dataset, has learned generic low-level features such as edges and textures, which are transferable and valuable for rolling bearing fault diagnosis. Freezing the Conv1 layer and the first three residual layers not only prevents overfitting on small datasets but also reduces computational overhead. Additionally, this strategy allows the network to focus on high-level feature extraction, improving both training efficiency and diagnostic accuracy.

Building upon previous studies, this research adopts a transfer learning strategy by freezing the Conv1 layer and the first three residual layers of ResNet-50 while transferring pre-trained ImageNet parameters to the ResCAA-ViT model. By fully leveraging the advantages of transfer learning, this study aims to enhance the accuracy and generalization capability of rolling bearing fault diagnosis models, providing a more reliable solution for fault identification under variable operating conditions.

## 3. Fault Diagnosis Model

**Figure 3:**
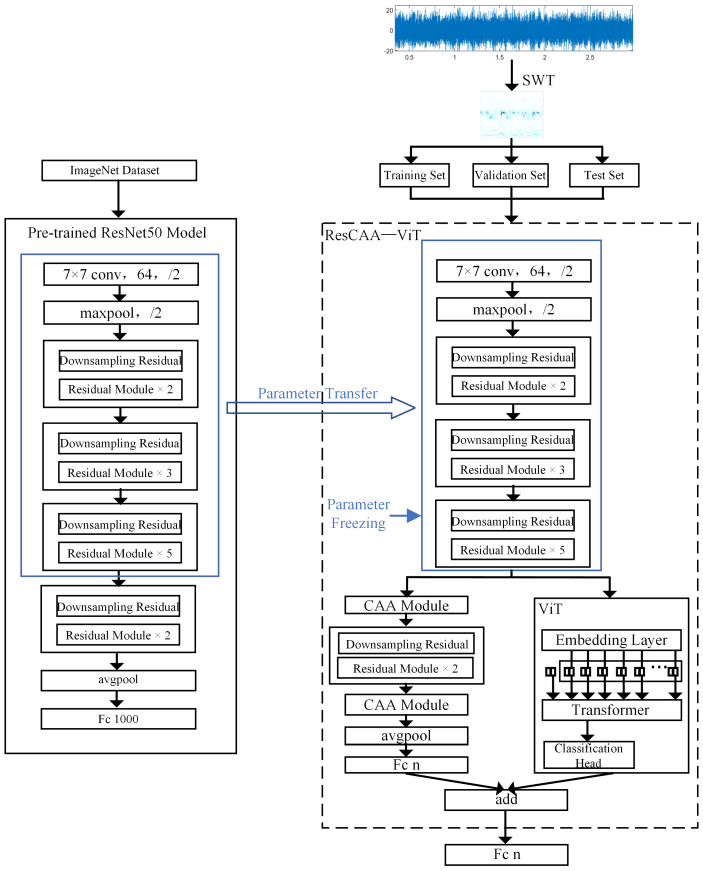
Proposed method network architecture.

This paper presents a method for fault diagnosis in rolling bearing, integrating the Synchronized Compressed Wavelet Transform (SWT) and the ResCAA-ViT architecture. The ResCAA-ViT network is an enhanced version of the ResNet-50 and Vision Transformer (ViT) networks. The architecture of the proposed method is shown in Figure 3.

First, the vibration signal is processed using the Synchrosqueezed Wavelet Transform (SWT) to generate a high-resolution time–frequency map, which is then input into the ResCAA-ViT model. The model’s convolutional and residual modules leverage parameters transferred from the pre-trained ResNet50. Next, the feature information output from the third residual module is directed into a dual-branch architecture for further processing. The left branch employs a residual network with the Context Anchor Attention (CAA) mechanism, aiming to fully utilize feature information from different layers. The CAA module in the early layers captures relationships between low-level features, while the CAA module in the later layers enhances the contextual modeling of high-level features, enabling the network to effectively extract both local and global features. Additionally, the CAA module integrates local feature extraction, strip convolution, and attention mechanisms, thereby enhancing the network’s focus on important fault features and improving classification accuracy and robustness. The right branch utilizes the Vision Transformer (ViT) model, which captures global features through a self-attention mechanism and leverages its global receptive field to better capture complex time–frequency features in the spectrogram. The outputs from both branches are combined through an addition operation, and the final diagnosis is performed using a Softmax classifier. The loss function combines cross-entropy loss and mean squared error (MSE) loss to improve the model’s consistency and collaborative learning capability. This hybrid architecture takes advantage of convolutional neural networks’ ability to efficiently extract local features, while the CAA attention mechanism improves the modeling of local feature contexts. In combination with the Transformer’s capacity to capture global dependencies, it significantly boosts the model’s ability to identify intricate fault characteristics.

The procedure for bearing fault diagnosis using the combination of SWT and ResCAA-ViT is outlined as follows:

(1) Sliding Window Sample Augmentation

The sliding window method generates multiple overlapping subsequences by applying a fixed-size window over the raw vibration signal and sliding it with a defined step size. This process augments the number of samples for model training, improving the model’s generalization ability and classification performance.

(2) SWT to Generate Time–Frequency Maps

For the augmented data samples of different fault types from step (1), the SWT is employed to produce high-resolution time–frequency representations. The images are then resized to 224 × 224 pixels. The time–frequency maps are randomly split into training, validation, and test sets with a ratio of 6:2:2.

(3) Model Training

The training set samples from step (2) are input into the ResCAA-ViT model. The parameters of the Conv1 layer and the first three residual layers are frozen, utilizing parameters pre-trained on the ImageNet dataset for the ResNet-50 model. Forward propagation is then applied to compute the predicted output, followed by backpropagation, which iteratively updates and optimizes the weights of the unfrozen layers. Finally, the trained network model is saved.

(4) Model Testing and Application

The test set samples from step (2) are fed into the trained ResCAA-ViT model, which then generates the fault diagnosis results. The classification accuracy of these test set samples is used to evaluate the model’s performance in fault diagnosis.

## 4. Experimental Results and Analysis

The experiments in this study were conducted using the Case Western Reserve University (CWRU) bearing dataset [24] and a real-world dataset from the non-drive end bearing of wind turbines at a wind farm. The experimental hardware configuration included an AMD EPYC 9754 128-Core Processor with 18 vCPUs, 60 GB of RAM, and a single RTX 3090 (24 GB) GPU. The experiments were carried out using Python (version 3.8) within the PyTorch (version 2.0.0) deep learning framework. The final results presented in this paper are based on the average of 10 test runs.

### 4.1. Case 1: CWRU Bearing Dataset Experiment

#### 4.1.1. CWRU Bearing Dataset and Preprocessing

**Figure 4:**
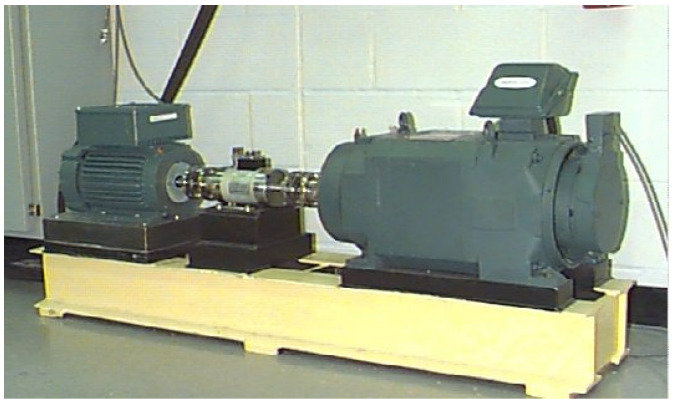
CWRU experimental setup.

The experimental setup at Case Western Reserve University (CWRU) is depicted in Figure 4. In this study, acceleration signal data were acquired through a drive-end acceleration sensor, with a sampling frequency set at 12 kHz. Performance evaluation was conducted under three distinct motor load conditions (0 hp, 1 hp, and 2 hp), which are represented by datasets A, B, and C.

The dataset consists of nine different fault types (such as rolling element, inner race, and outer race faults) along with one normal condition, each featuring damage diameters of 7 in, 14 in, and 21 in. A sliding window approach for sample augmentation was applied, with a window size of 1024 and a sliding step of 240. To ensure sufficient data, each fault type was expanded to 500 samples, and the corresponding sample labels are listed in the table. The experimental dataset is shown in Table 1.

**Figure 5:**
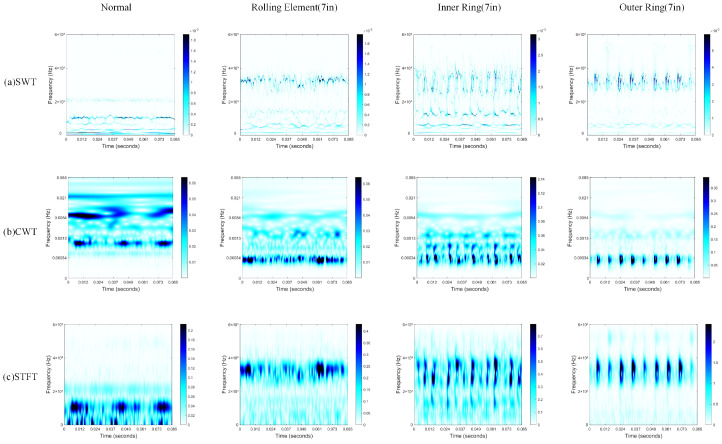
Comparison of time–frequency maps generated by different time–frequency analysis methods.

In this research, the Morlet wavelet was selected as the basis function for implementing the Synchrosqueezed Wavelet Transform (SWT). Figure 5 presents the time–frequency representations generated by different classical time–frequency analysis methods, including the Continuous Wavelet Transform (CWT), Short-Time Fourier Transform (STFT), and SWT, for normal, rolling element (7 in) fault, inner ring (7 in), and outer ring (7 in) data samples under operational conditions. Compared to the CWT and STFT, the time–frequency representations produced by the SWT exhibit more concentrated energy distribution and higher time–frequency resolution. This high-resolution time–frequency map is input into the ResSE-ViT network model designed in this study. The enhanced resolution allows the ResSE-ViT model to better capture both local and global features associated with the faults, improving fault classification accuracy and robustness under varying operational conditions.

#### 4.1.2. Experimental Parameter Settings

In this experiment, the learning rate was initially set to 0.0001, a commonly used value in Transformer model training. To evaluate its impact, different learning rates (0.001, 0.0005, 0.0001, and 0.00001) were tested. The results demonstrated that a learning rate of 0.0001 resulted in the best performance in terms of fault classification accuracy and convergence. Therefore, a learning rate of 0.0001 was chosen for the final model training, and the Adam optimizer was employed for adaptive optimization throughout the training.

The experiment was conducted with varying batch sizes of 8, 16, 32, and 64, while keeping all other experimental parameters constant. The results are presented in Table 2. The experimental groups labeled A—A/B/C refer to using the training set of dataset A for model training, while the test sets of datasets A, B, and C were used for testing. The average testing accuracy for these groups is provided in the table. Based on the results, a batch size of 16 was selected for model training, as it exhibited superior diagnostic accuracy compared to the other batch sizes.

**Figure 6:**
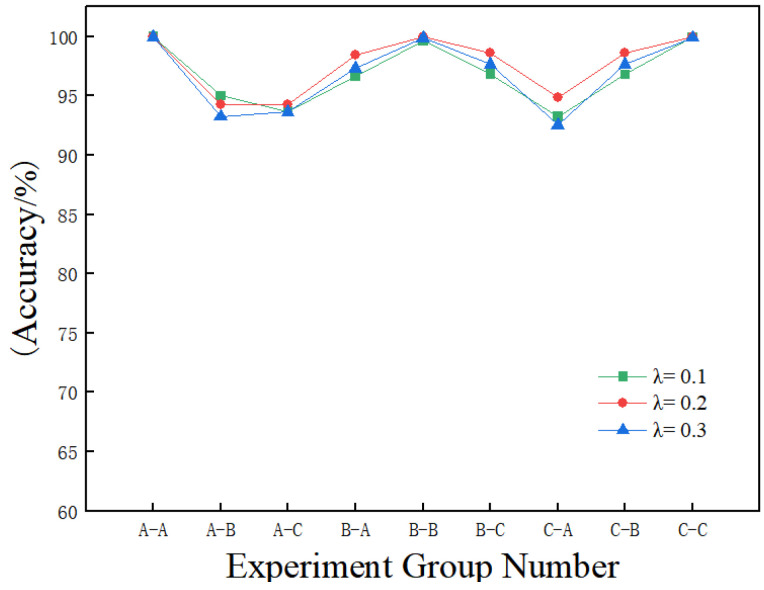
Fault diagnosis accuracy for different weight values in different experimental groups.

Figure 6 illustrates the fault diagnosis accuracy of the proposed method across different experimental groups, with varying values of the weight parameter λ in Equation (2). The experimental group labeled A-B indicates that dataset A was used for training, while dataset B served as the test sample, with other groups defined similarly. Analysis of the test set accuracy revealed that the method performed consistently well for different values of λ under constant operating conditions. However, the best fault diagnosis performance under different operating conditions was observed when λ was set to 0.2. As a result, the value of λ in the loss function was fixed at 0.2.

#### 4.1.3. Experimental Results and Performance Analysis

(1) Analysis of Constant Load Experiment

As shown in Figure 6, when the value of λ is set to 0.2, the proposed method achieves average prediction accuracies of 99.98%, 99.95%, and 99.94% in the constant operating condition experimental groups A-A, B-B, and C-C, respectively. These results demonstrate that the proposed method effectively classifies various fault states of the bearing.

**Figure 7:**
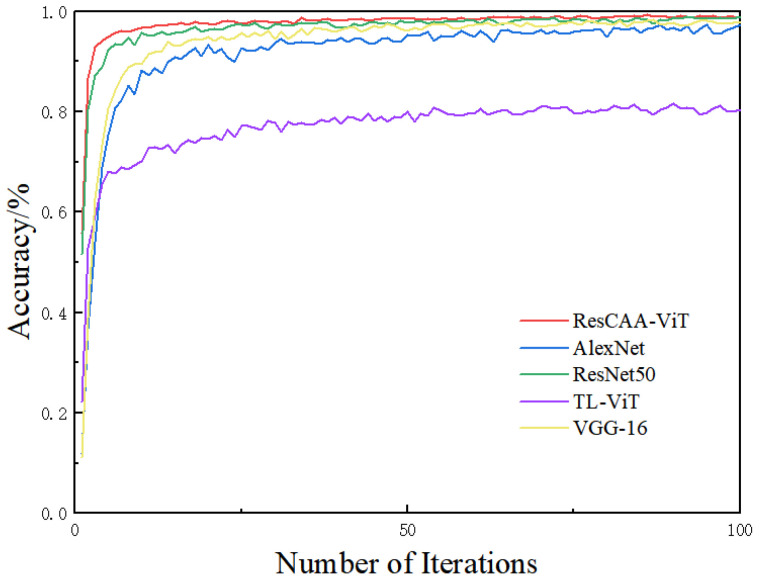
Training set accuracy of different models during training.

In order to further assess the benefits of the proposed algorithm, dataset A was used as an example, with the time–frequency maps generated by the SWT fed as input to the model. The proposed ResCAA-ViT model was compared with four widely-used deep learning models from the computer vision domain (AlexNet, ResNet50, TL-ViT, and VGG-16). Among them, the TL-ViT model employed transfer learning using pre-trained ViT model parameters from the ImageNet dataset, fine-tuning only the parameters of the final fully connected layer. Figure 7 displays the training set accuracy during the training process for each model, highlighting the superior performance of the proposed method. Through the use of transfer learning with the pre-trained ResNet50 model, the training and optimization process was expedited, reaching more than 99% accuracy on the validation set after only two epochs.

**Figure 8:**
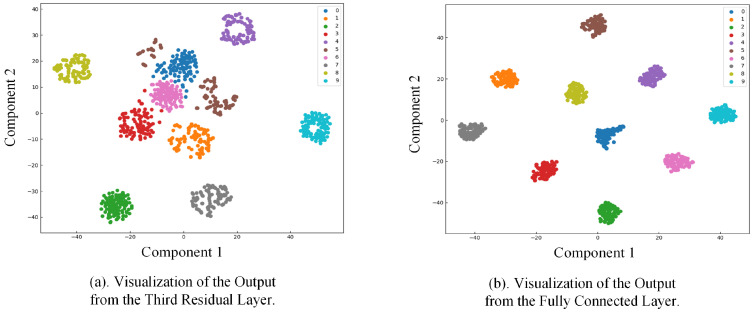
Visualization of model outputs.

To offer a clearer visualization of the feature extraction capabilities of the proposed method, Dataset A was chosen for demonstration. The t-SNE (t-distributed Stochastic Neighbor Embedding) dimensionality reduction algorithm was applied to map the high-dimensional features from the third residual layer of ResCAA-ViT and the final fully connected layer into two dimensions, as depicted in Figure 8.

As depicted in Figure 8a, after processing through the first three residual layers, the data samples are largely separated by class, although some samples are not yet fully clustered. To further optimize the feature representations, the samples were passed through the dual-branch network architecture. The left branch enhances the feature extraction capability of the residual network by integrating the CAA attention mechanism. This integration effectively improves the model’s focus on critical regions through efficient contextual modeling and local feature extraction. Meanwhile, the right branch leverages the Transformer model to capture global dependencies. Figure 8b illustrates the output from the final fully connected layer, where samples from different classes are distinctly clustered in the two-dimensional space, with the fault types being accurately classified.

(2) Analysis of Variable Load Experiment

**Figure 9:**
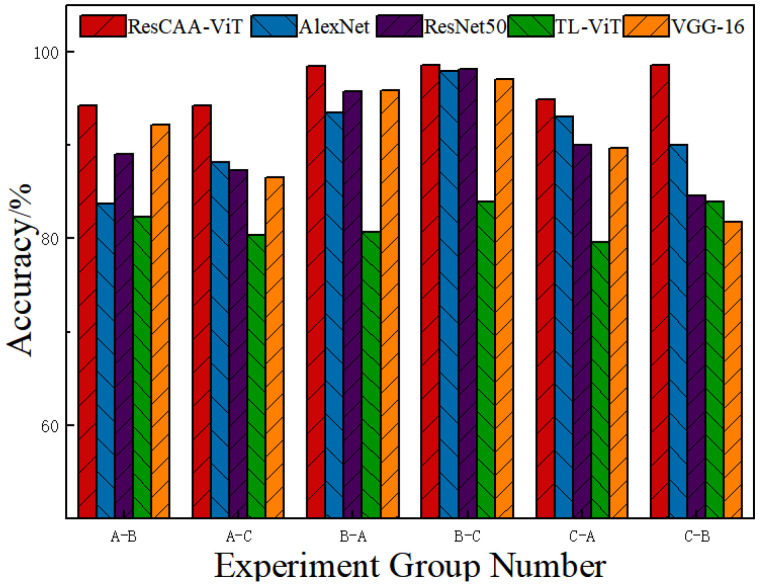
Experimental results of different network models under variable load conditions.

To assess the generalization capability of the proposed method, datasets A, B, and C, corresponding to three different loads, were employed. One dataset was designated for training and validation, while the other two datasets were used for testing. Figure 9 illustrates the experimental results, which compare the performance of the previously mentioned deep learning models with the ResCAA-ViT network model.

Figure 9 illustrates that the proposed method achieves bearing fault diagnosis accuracies surpassing 94% across different load conditions, with an average accuracy of 96.51%. This result outperforms other methods, highlighting the robust generalization ability of the approach under variable load conditions. The CAA attention mechanism enhances the representation of important features, where the CAA module improves focus on key regions through the synergistic effects of local feature extraction and contextual modeling. Additionally, the Transformer captures global dependencies, while the transfer-learned ResNet50 benefits from its strengths in local feature extraction. Compared to single-architecture models such as AlexNet, ResNet50, TL-ViT, and VGG-16, the proposed method exhibits superior feature extraction and generalization performance.

**Figure 10:**
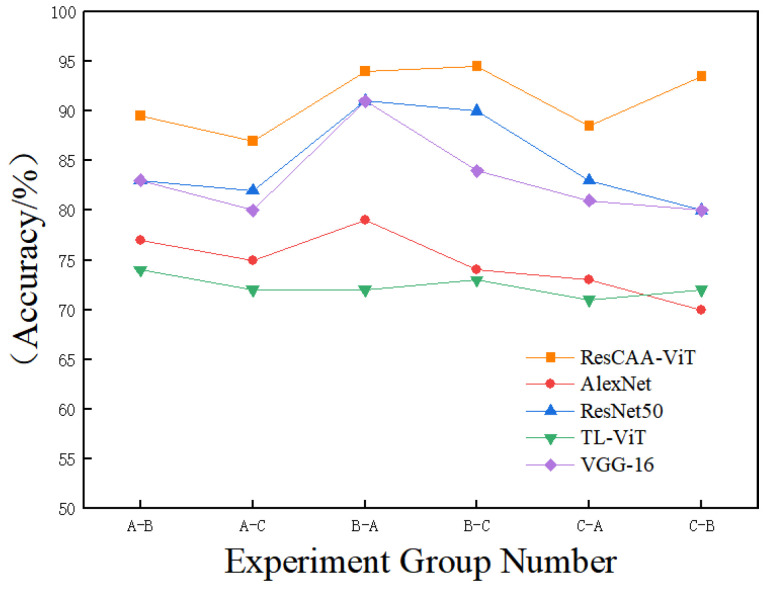
Fault diagnosis results for each group under 9 dB signal-to-noise ratio conditions.

In industrial applications, the operating conditions of rotating machinery are often intricate, characterized by variable loads and considerable noise interference. To evaluate the noise robustness and generalization capability of the proposed method, three distinct load conditions were established, with one used for training and the others reserved for testing. Additionally, noise with a signal-to-noise ratio of 9 dB was introduced into the test set for validation purposes. The experimental outcomes are depicted in Figure 10.

As demonstrated in Figure 10, under both noisy and variable load conditions, the proposed ResCAA-ViT method consistently outperforms the four comparison models in terms of classification accuracy for bearing fault diagnosis. At a 9 dB signal-to-noise ratio, the accuracy ranges from 88.5% to 94.5%, highlighting the model’s robust classification performance in complex operational environments and its distinct advantage over the comparative models. The results further demonstrate that the ResCAA-ViT model surpasses traditional convolutional neural networks in feature extraction. By integrating the synchronized wavelet transform (SWT) with the enhanced Vision Transformer (ResCAA-ViT) architecture, the model effectively captures instantaneous changes and local features in vibration signals, achieving significantly higher time–frequency resolution than conventional methods. Additionally, the incorporation of the CAA attention mechanism alongside the Vision Transformer’s self-attention mechanism enables the model to accurately identify key features associated with bearing faults while mitigating the influence of noise on diagnostic outcomes. These results validate the model’s powerful feature extraction capabilities and classification performance, emphasizing its strong potential for bearing fault diagnosis in complex conditions and its promising application prospects in industrial engineering.

(3) Comparison of different time–frequency analysis methods

**Figure 11:**
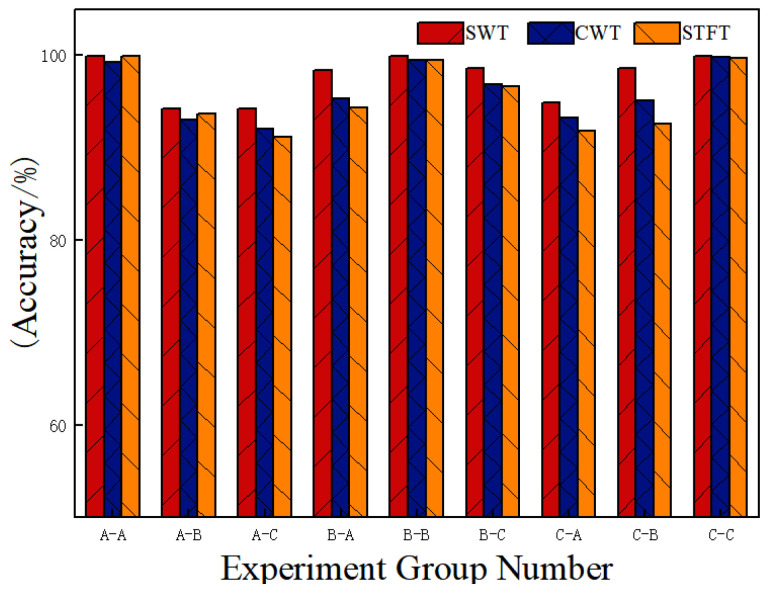
Experimental results of different time–frequency analysis methods under variable load conditions.

To evaluate the effectiveness of the SWT-based time–frequency analysis method proposed in this study, time–frequency representations generated using SWT, CWT, and STFT were utilized as inputs to the ResCAA-ViT network model. Comparative experiments were performed under variable load conditions, and the results are presented in Figure 11.

As shown in Figure 11, the classification accuracies of the three methods are comparable under constant load conditions. However, under varying load conditions, the SWT method significantly outperforms both the STFT and CWT methods. Unlike the CWT and STFT time–frequency analysis methods, the SWT generates time–frequency maps with more concentrated energy and higher resolution, enabling better capture of instantaneous changes and local features in the signal. These findings indicate that high-resolution time–frequency maps produced by SWT, when used as inputs to the network model, effectively enhance the model’s generalization ability.

(4) Ablation Experiment

**Figure 12:**
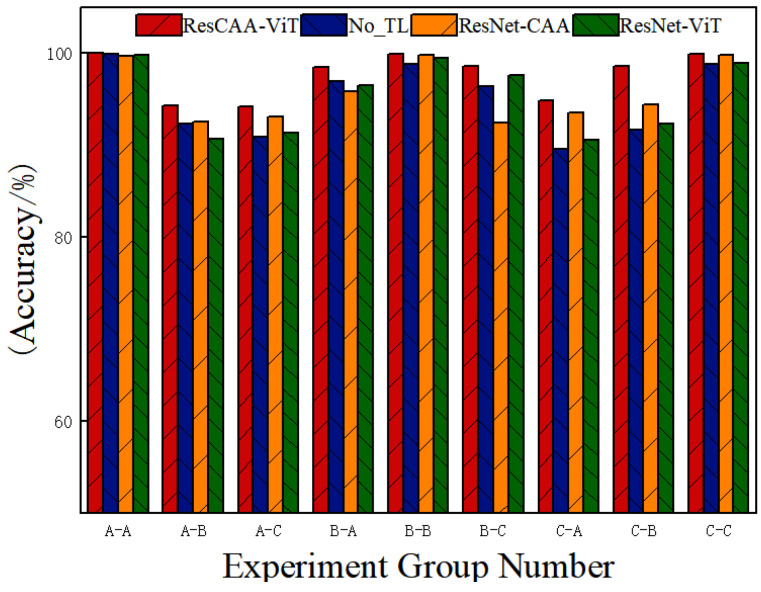
Ablation experiment.

To systematically evaluate the impact of different network components on fault diagnosis performance, we conducted ablation experiments to analyze the role and contribution of each module to the model’s overall efficiency. The experiments compared four network architectures under both constant and variable load conditions: (1) the proposed ResCAA-ViT model; (2) a ResCAA-ViT model without pre-trained transfer learning (NO_TL); (3) the ResNet-CAA model, which includes only the main and left branches; and (4) the ResNet-ViT model, which consists of only the main and right branches. The results are illustrated in Figure 12.

As shown in Figure 12, under constant load conditions, the ResCAA-ViT model achieves slightly better performance than the comparison models. However, under variable load conditions, its superiority becomes more evident, significantly outperforming the other architectures. Specifically, the proposed ResCAA-ViT model achieves the highest average diagnostic accuracy (96.51%), surpassing the performance of the single-branch models (95.12% for ResNet-CAA and 95.26% for ResNet-ViT) as well as the NO_TL model (94.03%). These results highlight the advantages of the dual-branch architecture over single-branch approaches, particularly in variable load scenarios. The NO_TL model, which lacks pre-trained initialization, requires more training epochs to reach competitive performance. However, due to the limited amount of training data, it struggles to fully capture discriminative fault features, resulting in suboptimal learning dynamics. The ResNet-CAA model (left branch only) benefits from convolutional layers and the CAA attention mechanism for local feature extraction. However, the absence of a global representation limits its adaptability to fluctuating operating conditions. Similarly, the ResNet-ViT model (right branch only) captures long-range dependencies via the self-attention mechanism, yet it struggles to preserve fine-grained local fault information, leading to decreased diagnostic performance in dynamic environments.

In contrast, the dual-branch ResCAA-ViT model effectively integrates both local and global features, significantly enhancing its adaptability to varying operating conditions. The left branch leverages CAA attention and convolutional layers to refine local feature representation, while the right branch utilizes ViT to capture global dependencies. Additionally, the incorporation of multi-loss constraints reinforces model consistency and collaborative learning. This hybrid architecture not only retains the advantages of convolutional networks in local feature extraction but also enhances focus through the CAA attention mechanism. Meanwhile, the Transformer-based right branch ensures robust global feature modeling, leading to an effective fusion of local and global features. In scenarios characterized by limited data and variable load conditions, the proposed model demonstrates superior classification accuracy and robustness, validating the effectiveness of its dual-branch design and transfer learning strategy.

### 4.2. Case 2: Experiment with the Non-Driven End Bearing Dataset of the Wind Turbines

**Figure 13:**
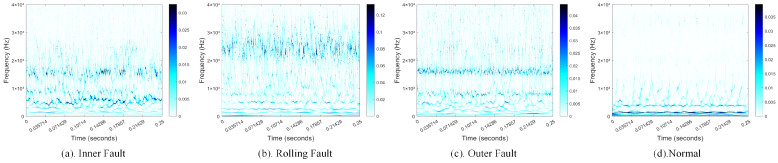
Time–frequency representations of different fault states obtained using SWT.

To evaluate the practical applicability of the algorithm proposed in this study, this section presents experimental validation and analysis based on real-world data collected from the non-drive end bearing of a 1.5 MW doubly-fed wind turbine generator located at a wind farm. The vibration signals were captured by an axial acceleration sensor at the non-drive end of the generator, with a sampling frequency of 16,384 Hz. Data were gathered for three distinct fault types of the non-drive end bearing—inner race fault, rolling element fault, and outer race fault—as well as for a normal state. Each sample consists of 4096 data points, with 1000 samples collected per fault type, yielding a total of 4000 data samples. The dataset was randomly partitioned into training, validation, and test sets at a ratio of 6:2:2, as detailed in Table 3. To extract informative features, a set of samples from each state was transformed using the Synchro-Compressed Wavelet Transform (SWT), resulting in high-resolution time–frequency representations. As shown in Figure 13, these transformations reveal distinct patterns for each fault type.

**Figure 14:**
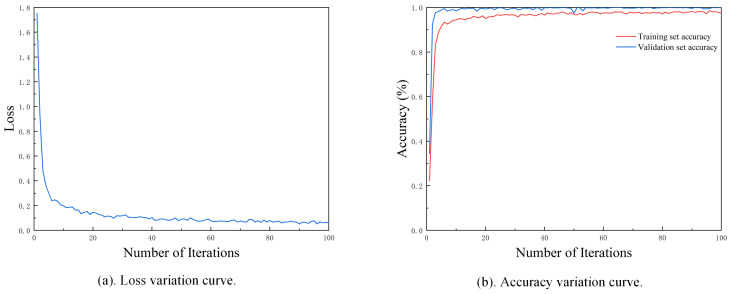
Loss and accuracy variation curves.

The time–frequency maps generated by the SWT continued to serve as the input for the model. The initial parameter settings for the experiments in this section were kept consistent with those used in Case 1. The model was trained for 100 epochs to ensure sufficient convergence. As shown in Figure 14a,b, the loss variation curve demonstrates a gradual decline, indicating effective learning and optimization of the model. Meanwhile, the accuracy variation curve exhibits a steady increase, eventually reaching a plateau, which suggests that the model achieves satisfactory fault classification performance with stable generalization.

**Figure 15:**
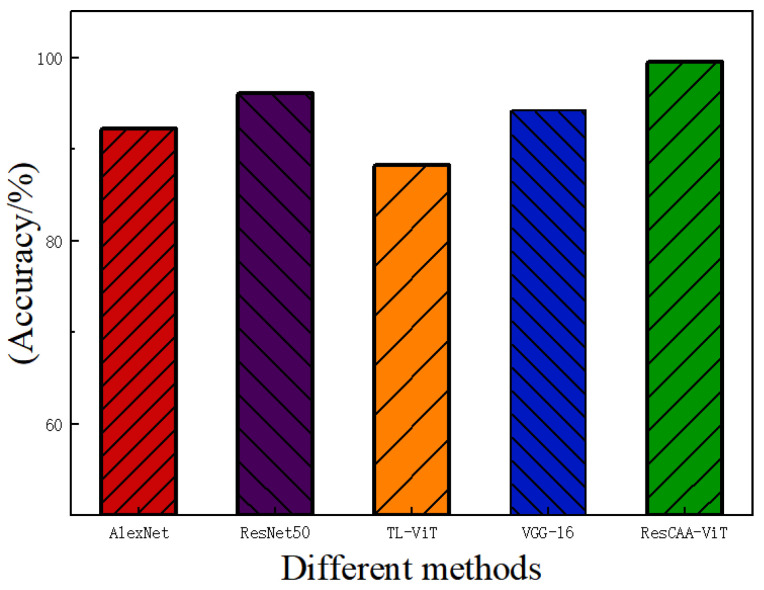
Fault diagnosis results of different network models.

Similar to Case 1, the experiment involves comparing the performance of four deep learning models—AlexNet, ResNet50, TL-ViT, and VGG-16—along with the ResCAA-ViT model proposed in this study. To ensure the reliability and consistency of the results, ten independent repetitions of the experiment were conducted, with each training set undergoing 100 epochs. Figure 15 presents the average classification accuracy of each model on the test set. The results indicate that the proposed ResCAA-ViT model outperforms all other models, achieving an impressive average accuracy of 99.25%, significantly exceeding the performance of the comparison models.

**Figure 16:**
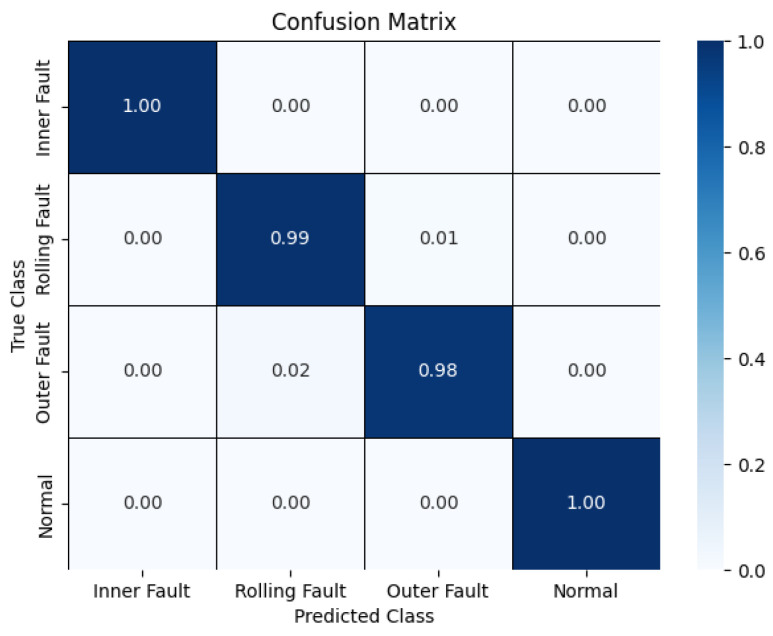
Confusion matrix.

To provide a comprehensive visualization of the ResCAA-ViT model’s classification performance across various fault categories, a confusion matrix is presented in Figure 16 to support the analysis. The results indicate a strong alignment between the model’s predictions and the actual labels, with only a limited number of misclassified samples. These experimental findings indicate that the proposed algorithm exhibits excellent fault classification capabilities when applied to the real-world data from the non-drive end bearing of a wind turbine generator, further confirming its substantial potential for industrial applications.

## 5. Conclusions

This paper presents a fault diagnosis approach for rolling bearings utilizing the SWT in combination with the ResCAA-ViT network, demonstrating its effectiveness under complex and variable operating conditions.

The method utilizes the SWT to transform vibration signals into high-resolution time–frequency maps. Compared to time–frequency maps generated by the CWT and STFT, the SWT more effectively captures instantaneous changes and local features in the signal, which enhances fault diagnosis performance when processed by the network model.

The ResCAA-ViT model introduced in this study combines the advantages of convolutional neural networks (CNNs) for extracting local features with the capability of Transformers to model global dependencies effectively. By incorporating transfer learning with a pre-trained ResNet50 model, the method addresses the challenges of limited data and accelerates model training and optimization. Furthermore, the CAA attention mechanism improves the model’s ability to represent features effectively, while the multi-loss constraint method improves consistency and collaborative learning.

Validation on the CWRU dataset demonstrates that the proposed method achieves average accuracies of 99.96% under constant load and 96.51% under variable load conditions, outperforming other methods. Additionally, the average diagnostic accuracy on the real-world dataset of the non-drive end bearing of wind turbines is 99.25%, which surpasses that of the comparison methods. These results highlight the high accuracy and strong generalization ability of the proposed method, making it suitable for real-world industrial applications. 

## Figures and Tables

**Table 1:** Experimental data.

Dataset	Load (HP)	Fault Type	Damage Diameter (in)	Class Label
A/B/C	0/1/2	Rolling Element	7	0
			14	1
			21	2
		Inner Ring	7	3
			14	4
			21	5
		Outer Ring	7	6
			14	7
			21	8
		Normal	0	9

**Table 2:** Average testing accuracy for different batch sizes across experimental groups.

Batch Size	A—A/B/C (%)	B—A/B/C (%)	C—A/B/C (%)	Average Accuracy (%)
8	94.53	95.12	95.89	95.18
16	96.16	96.39	96.98	96.51
32	94.23	94.75	95.33	94.77
64	92.91	93.35	94.12	93.46

**Table 3:** Sample distribution for generator non-drive end bearing fault types.

Generator Non-Drive End Bearing State	Label	Training Samples	Validation Samples	Testing Samples
Inner Fault	0	600	200	200
Rolling Fault	1	600	200	200
Outer Fault	2	600	200	200
Normal	3	600	200	200

## Data Availability

The dataset is available on request from the authors.
